# Supermarkets in Cyberspace: A Conceptual Framework to Capture the Influence of Online Food Retail Environments on Consumer Behavior

**DOI:** 10.3390/ijerph17228639

**Published:** 2020-11-20

**Authors:** Neha Khandpur, Laura Y. Zatz, Sara N. Bleich, Lindsey Smith Taillie, Jennifer A. Orr, Eric B. Rimm, Alyssa J. Moran

**Affiliations:** 1 University of São Paulo, Av. Dr. Arnaldo, 715 Cerqueira César, São Paulo SP 01246-904, Brazil; 2 Harvard T.H. Chan School of Public Health, Harvard University, 677 Huntington Ave, Boston, MA 02115, USA; laz491@mail.harvard.edu (L.Y.Z.); sbleich@hsph.harvard.edu (S.N.B.); erimm@hsph.harvard.edu (E.B.R.); 3 Gillings School of Global Public Health, University of North Carolina at Chapel Hill, 135 Dauer Dr, Chapel Hill, NC 27599, USA; taillie@unc.edu; 4 University of Pennsylvania, 423 Guardian Drive, Philadelphia, PA 19104-6021, USA; Jennifer.Orr@pennmedicine.upenn.edu; 5 Bloomberg School of Public Health, Johns Hopkins University, Baltimore, MD 21218, USA; amoran10@jhu.edu

**Keywords:** online food retail, conceptual framework, consumer behavior, food choices, online shopping, retailer policies

## Abstract

The rapid increase in online shopping and the extension of online food purchase and delivery services to federal nutrition program participants highlight the need for a conceptual framework capturing the influence of online food retail environments on consumer behaviors. This study aims to develop such a conceptual framework. To achieve this, mixed methods were used, including: (1) a literature review and development of an initial framework; (2) key informant interviews; (3) pilot testing and refinement of the draft framework; and (4) a group discussion with experts to establish content validity. The resulting framework captures both consumer- and retailer-level influences across the entire shopping journey, as well as the broader social, community, and policy context. It identifies important factors such as consumer demographic characteristics, preferences, past behaviors, and retailer policies and practices. The framework also emphasizes the dynamic nature of personalized marketing by retailers and customizable website content, and captures equity and transparency in retailer policies and practices. The framework draws from multiple disciplines, providing a foundation for understanding the impact of online food retail on dietary behaviors. It can be utilized to inform public health interventions, retailer practices, and governmental policies for creating healthy and equitable online food retail environments.

## 1. Introduction

Online food retail is an increasingly popular means of acquiring food and is expected to grow rapidly over the coming decade. In 2017, online food retail represented $13 billion in sales [1] and was projected to increase to $100 billion [2], reaching 70% of U.S. shoppers by 2025 [3]. The 2019 coronavirus (COVID-19) pandemic has accelerated this growth and served as a catalyst for retailers to increase investments in their online food retail infrastructure and services. In April 2020, U.S. shoppers spent $5.3 billion on online food purchases, an increase of 37% from the previous month [4,5]. Based on this recent surge, revised growth projections estimate a nine-fold increase in online grocery purchases between 2017 and 2023 [6].

Online food retail has also emerged as an important avenue to improve food access. In 2018, the United States Department of Agriculture (USDA) selected 10 retailers in 9 states for a two-year Online Purchasing Pilot (“online Electronic Benefits Transfer (EBT)”) to test the use of Supplemental Nutrition Assistance Program (SNAP) benefits as payment for online food purchases [7]. The affordability, access, and delivery inequities, as well as the disproportionate food security challenges faced by low-income communities have been brought to the fore during the pandemic [8], prompting the USDA to expand online EBT both geographically and across a wider range of retailers. At the time of writing, at least 40 states had been approved to participate in online EBT, with several others in the planning stage [9]. The Special Supplemental Nutrition Program for Women, Infants, and Children (WIC) is also considering how to offer online food purchasing options to its participants [10].

These shifts in consumer food purchase behaviors, the increasing investment in online infrastructure, and the expansion of online food purchasing to participants in federal programs, highlight the need for an assessment of the online food retail environment. A substantial and growing body of evidence captures the influence of in-store food marketing on consumer purchases [11,12]. In comparison, little is known about the relationship between the online food retail environment and consumer food choices. Some evidence supports the influence of social, contextual, and demographic factors on consumers’ use of online platforms for food purchases [13,14,15]. Retailer-level factors like credibility, product freshness, product quality and price have also been shown to predict online purchasing [16]. However, there is a dearth of information about factors influencing the spectrum of online consumer behaviors which is important for understanding how online food retailers influence consumer food purchases and subsequent dietary intake and health outcomes.

What is currently lacking is an integrated framework capturing both consumer- and retailer-level factors and their interaction that influence consumer behaviors within online environments. The few existing frameworks focus on specific consumer determinants like their attitudes, privacy concerns, social influences, facilitating conditions, hedonic motivations, and perceived risk or satisfaction with the online experience [17,18,19]. Retailer influence is incompletely captured or mentioned briefly [20]. The models do not illustrate the interactive and dynamic nature of online food retail platforms or the active and responsive role that consumers play in shaping their food purchase experience. Existing frameworks are also explicitly geared towards retaining and expanding the consumer base or maximizing profits [21]; they are not designed to study the effects these platforms have on dietary behaviors or health. Perhaps most revealing is the concentration of this literature in the fields of marketing, retail, decision sciences, and informatics; studies are largely absent in the public health domain.

In the absence of a comprehensive conceptual framework that looks at consumer grocery purchase behaviors it becomes impossible to systematically study the effect of food retail environments on food choices. Such a framework is crucial for informing public health interventions, guidelines, retailer practices, and governmental policies to create healthy and equitable online food retail environments. To address this gap in the literature, the present study aims to develop and refine a conceptual framework capturing factors that influence consumer food purchase behaviors within online food retail environments. For the purposes of this study, online food retail environments were described as websites providing click-and-collect (i.e., order online and pick up at the store) or food delivery services. ‘Retailers’ include e-commerce platforms hosted by the retailer themselves or by a third-party vendor (e.g., Instacart).

## 2. Materials and Methods

The development and refinement of the conceptual framework was guided by the approach suggested by Jabareen, 2009 [22], and involved extensive reading and categorizing of data; identifying concepts; deconstructing, categorizing, synthesizing, and integrating concepts; and validating the final framework. To achieve this, mixed methods were used, including:

1. A literature review and development of an initial framework
2. Key informant interviews
3. Pilot testing and refinement of the draft framework
4. Group discussion with experts to establish content validity

The study methodology was reviewed and determined to be non-human subjects research by the Institutional Review Board at the Johns Hopkins Bloomberg School of Public Health.

### 2.1. Literature Review

A scoping review was undertaken between April and June of 2019 to identify peer-reviewed and grey literature, in English, on the attributes, preferences, and shopping behaviors of consumers that make purchases online, consumer interaction with and acceptance of technology, online and in-store food marketing and merchandising, and design of online retail environments. Databases were searched from January 2009 to May 2019 (ProQuest, PubMed, and Thomson ONE) using combinations of the keywords food, grocer*, supermarket*, retail*, shop*, store*, purchas*, buy*, online, ecommerce (or e-commerce), internet, and *web*. Search results were supplemented with health agency reports, trade publications, and industry reports from 2015 to 2019. Reference lists from peer-reviewed publications were also scanned. While peer-reviewed literature was not restricted by geographic location, only US-focused analyst reports and trade publications were included to ensure contextual relevance. Paper titles and abstracts were screened. A total of 136 industry reports and 97 peer-reviewed papers informed the development of the draft framework.

### 2.2. Key Informant Interviews

We interviewed seven experts in grocery merchandizing and marketing, e-commerce and online retail, behavioral psychology, public policy, computer science and data privacy and digital marketing. Experts were identified through a combination of known contacts, through their published work, or were referred to by other experts during interviews. The interviews were conducted in person or via teleconference by a member of the research team and lasted 45–60 min.

The interview began with an overview of the objectives of the study. Experts were presented with the draft framework and asked: (i) for their feedback on the extent to which it captured their understanding of factors influencing consumer food choices when shopping online; (ii) to identify constructs that could be improved or simplified; and (iii) for additional constructs that should be included. Follow-up questions were tailored to each key informant’s area of expertise. For example, an expert in computer science and engineering was asked an additional question about when and how personal data are collected from consumers along the path to purchase. Experts in food retail marketing were asked how personal data are used to adapt online marketing practices to specific consumers. Insights were requested on how the key domains influenced one another. Suggestions were incorporated, and the framework was refined after each interview.

### 2.3. Pilot Testing

Study researchers (A.J.M., N.K.) independently tested the internal consistency of the conceptual framework by using it to guide a mock shopping exercise. This was done to identify additional concepts that may have been missed during the literature review and the key informant interview, but not with the aim of formally testing the framework. An online shopping account was created at two U.S. online food retailers. Researchers navigated each store’s website, browsed through their departments, added three grocery items to the shopping cart, and proceeded to the checkout. The applicability of the conceptual framework to the experience of a consumer searching for and selecting food was discussed in detail. Revisions to the framework were incorporated as necessary.

### 2.4. Expert Discussion

A teleconference discussion was convened with six members of the Healthy Food Retail Working Group to determine the content validity of the conceptual framework. This group is a collaborative effort of the Robert Wood Johnson Foundation’s Healthy Eating Research program and the Centers for Disease Control and Prevention’s Nutrition and Obesity Policy Research and Evaluation Network. Members include researchers and technical experts working on healthy food retail and related areas.

To engage fully with the framework, members of the Working Group were asked to undertake a mock shopping exercise, similar to the one conducted by the study authors, two weeks prior to the call. A Qualtrics form was created to guide the sequence of product searching and selection activities and to record feedback. After selecting an online food retailer from a list of 21 options, members navigated to the grocery department homepage, the breakfast cereal department page, and the product pages of two specific brands of bread and canned fish. They documented marketing strategies, customizable features, tools, options, and multimedia content on each of these webpages. Members also recorded ease of navigation and site policies. Their feedback from this exercise was discussed in the teleconference, during which the purpose of the research was clarified, and a draft of the conceptual framework (including what the concepts represent and how they relate to each other) was presented. Members were asked whether the current framework captured their understanding of the range of factors influencing consumer behavior in the online store and for ideas for improvement. Notes from the discussion were recorded, and relevant revisions were incorporated into the framework.

## 3. Results

### 3.1. Evolution of the Conceptual Framework

Insights from the literature review informed the development of a draft framework that captured the influence of consumer characteristics, online food marketing, and retailer and website characteristics on online grocery shopping intention. Feedback from the key informant interviews added further detail by delineating the different stages of the online shopping process, the sequence of online consumer behaviors, the key role of personalized marketing, relationships between online retailers and manufacturers, disclosure of sponsored content, factors influencing site design, and the use of personal information in customizing the platform.

The pilot test provided additional insights into retailer characteristics related to membership and loyalty programs, privacy and data use, and order payment, fulfillment, and collection. This exercise also differentiated the static versus the dynamic elements of the online platform (i.e., attributes of the site that are consistent for all consumers versus those that can be personalized by the retailer or customized by the consumer), differences in site functionality as viewed by an anonymous shopper versus a registered shopper, and marketing strategies employed at checkout.

Healthy Food Retail Working Group members acknowledged the detail and clarity of the current version of the framework and agreed that it captured relevant constructs that influence consumer food choice in online environments. Their feedback focused on the applicability of the conceptual framework to specific subgroups of consumers (e.g., SNAP and WIC beneficiaries), retailers (e.g., small stores), and online formats (e.g., mobile applications). These ideas were discussed, and relevant constructs in the framework were added or made more salient.

The final framework combined elements of the Technology Acceptance Model, consumer behavior and decision-making frameworks, brick-and-mortar marketing categories (product, price, placement, and promotion) [11,23], key themes from the data privacy and e-commerce literature, and constructs described in existing food environment measures (e.g., Nutrition Environment Measures in Stores) [18,20,24,25,26,27,28,29,30].

### 3.2. Description of the Conceptual Framework

#### 3.2.1. Path to Purchase

Central to the conceptual framework is the sequence of consumer behaviors involved in online grocery shopping. This Path to Purchase consists of four stages—Pre-Shop, Online Shopping, Pick-Up or Delivery, and Post-Shop—encompassing six behaviors. Under Pre-Shop behaviors, the consumer selects an online retailer. He/she then searches for or discovers, selects, and purchases food (Online Shopping). The consumer receives the order (Pick-Up or Delivery) and prepares and consumes the food or/and discards it (Post-Shop). The quality of the consumer’s experience engaging in each of these behaviors determines their overall satisfaction with the retailer and likelihood of shopping again.

**Figure 1:**
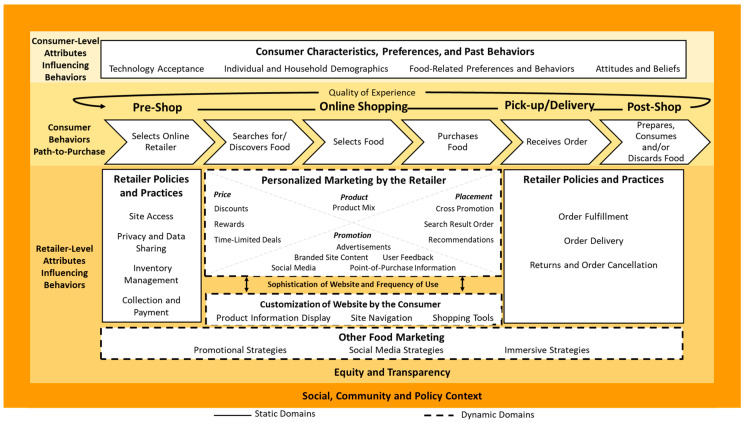
Consumer behavior in the online food retail environment.

Consumer behaviors are influenced by consumer- and retailer-level attributes, presented above and below the Path to Purchase in Figure 1. Attributes presented in solid outline are not likely to change over the shopping journey (static domains). Attributes presented in dashed outline are likely to change over the duration of a single shopping visit (dynamic domains). The cross-cutting themes of Equity and Transparency influence retailer-level factors, while Social, Community, and Policy Context influences both retailer- and consumer-level factors.

#### 3.2.2. Consumer-Level Attributes

Consumer Characteristics, Preferences, and Past Behaviors: this domain encompasses consumers’ Technology Acceptance, Individual and Household Demographics, Food-Related Preferences and Behaviors, and Attitudes and Beliefs (detailed in Table 1). These attributes may influence decision-making at each stage of food selection and purchase. For instance, consumers with positive experiences with e-commerce and online food retail may be more likely to select an online retailer. Individual and demographic characteristics such as age, education, income, household composition (e.g., having young children) and location also influence the likelihood of shopping online, the platform selected, the foods purchased, and their preparation and consumption.

#### 3.2.3. Retailer-Level Attributes

Retailer Policies and Practices: they include static attributes of online retailers that may influence retailer selection, food receipt, and food preparation and consumption. During retailer selection, Site Access policies include information required to purchase groceries online, retailer incentives offered to first-time users (e.g., free trials), membership requirements prior to shopping, and delivery service areas (e.g., zip codes served). Privacy and Data Sharing policies include the terms and conditions that govern the collection, tracking, storage, use, and sharing of personal information by the retailer. Policies on Inventory Management guide the availability of products and brands, pricing strategy, and accuracy of inventory tracking, while policies on Collection and Payment determine the payment options accepted by the retailer (e.g., SNAP), the integration of loyalty and reward programs, fees for delivery and restocking, and delivery options (e.g., availability of click and collect or delivery).

The Pick-Up and Post-Shop behaviors are likely to be determined by Retailer Policies and Practices that govern Order Fulfillment, Order Delivery, and Returns and Order Cancellation. Policies on Order Fulfillment include the price and appropriateness of product substitutions during stock-outs and the quality of the food received. The availability of convenient time slots, delivery coordination (e.g., text message communication, online order tracking), and the availability of secure delivery options are examples of policies associated with Order Delivery. Policies determining the ease of cancelling incomplete or incorrect orders of items purchased online (e.g., store credit, vouchers, full refund) relate to Returns and Order Cancellation.

Personalized Marketing by Retailers: a consumer’s search, selection and purchase of food are influenced by factors within the dynamic domain of Personalized Marketing. These marketing strategies are based on personal information provided by the consumer when registering with the online platform and from past purchases and browsing history. They may also be based on consumer data purchased by the retailer, shared by third parties, or automatically collected upon visiting the site (e.g., IP address, operating system). The consumer, either knowingly (by actively agreeing to) or unknowingly (by using the site, by signing-up for an account or a loyalty card that activates implicit agreements) or without full knowledge of the implications, allows retailers to use various sources of information to create a tailored experience.

Personalized Marketing maps onto the marketing mix of Product, Price, Placement, and Promotion (“the 4Ps”), but manifests differently in the online food retail environment than in brick-and-mortar stores [11]. Product-related personalized marketing includes Product Mix or the range and variety of products the consumer can view and explore. In the online space, this may include personalized storefronts created for the consumer which display products aligning with the consumer’s revealed preferences (e.g., vegan, gluten-free). Product Price relates to different types of Discounts, Rewards, or Time-Limited Special Deals offered. These strategies can be personalized (e.g., the discounted products or discount amount is tailored) and often interact with Placement or Promotional strategies (e.g., recommended products may also be discounted). Under Placement, Cross-Promotion describes suggested complementary products, Search Result Ordering describes the default appearance of products (e.g., higher cost or sponsored products may appear before other options), and Recommendations captures the strategies used to increase exposure to featured, seasonal, or popular products on the site—all of which can be personalized to the consumer’s preferences. Promotion refers to personalized marketing to increase consumer exposure to or visibility of sponsored products through Advertisements (e.g., title cards or banner advertisements), Branded Site Content, User Feedback (e.g., product ratings and reviews), links to Social Media, and other Point-of-Purchase Information (e.g., product labels). These strategies are frequently used in combination to influence product discovery, selection and purchase on the website homepage, search results page, product page, or at checkout.

Customization of the Website by the Consumer: this is the other dynamic domain that influences the search, selection, and purchase of food and includes Product Information Display, Site Navigation, and Shopping Tools. These attributes allow consumers to change what nutrition information they see (Product Information Display), filter products based on preferred attributes, save shopping lists, or request certain product comparisons (Shopping Tools). Combined with tools and tutorials to ease website navigation (Site Navigation), website customization features can increase the convenience of product searches, enhance consumer engagement with the product catalogue and improve the quality of the food purchase experience.

Other Food Marketing: this dynamic domain includes Promotional Strategies, Social Media Strategies, and Immersive Strategies, recognizing that exposure to marketing in other settings (brick-and-mortar stores), through direct-to-consumer promotions, product endorsements, and sponsorships, and targeted marketing through social media platforms will affect food choices made online.

Sophistication of Website and Frequency of Use: technological progress in interface design, communications, and data security is likely to improve consumer trust and increase the volume and frequency of purchases made online. Advancements in personal data collection, advertising technology, purchase data analytics, and consumers’ increased involvement in the co-creation of food retail platforms will allow retailers to better profile customers and more efficiently match them to products and promotions, increasing engagement. In this way, more sophisticated online platforms and frequent consumer visits will ensure greater personalization of retailer marketing strategies and a more customized website.

#### 3.2.4. Cross-Cutting Domains

Equity and Transparency are fundamental to retailer engagement with the consumer. Equity refers to the differential impact of retailer policies and practices on the food behaviors and privacy of underserved populations (e.g., individuals and communities of Color, low-income households, older adults, people with disabilities, households in rural areas). For example, a consumer’s ability to utilize online services may be affected by the retail service area, availability of convenient delivery slots, or accepted payment methods. A retailer’s targeted and personalized marketing strategies may trigger impulse purchases or increase the basket size, differentially impacting low-income consumers, especially if the promoted products are of inferior nutritional quality. Transparency in policies and practices captures the retailer’s clear and upfront disclosure of data collection, storage and use of data, surveillance methods, marketing, sponsorships, etc., that may consciously or unconsciously influence the consumer’s choices along the Path to Purchase. For instance, disclosure of fees and hidden costs and collection of personal data prior to checkout may affect a consumer’s choice of retailer. Disclosure of product sponsorship at point of purchase may affect product selection.

Social, Community, and Policy Context: this conceptual framework is nested in the socio-ecological model [31]. Consumer and Retailer-Level attributes are likely influenced by the social context (e.g., social norms, endorsements from trusted members of society) and the structural factors (community, institutional, and policy contexts) in which they are embedded. The surge in online grocery purchases resulting from the physical distancing measures implemented in 2020 is an example of how contextual factors can affect consumers behaviors. Similarly, the acceptance of SNAP benefits for online food purchases would first require a favorable state-level policy context (e.g., states need approval for use of EBT test cards) [7], before retailer policies can be implemented. In this way, the provision of certain services by retailers is very much dependent on the broader political context.

## 4. Discussion

This study presents a conceptual framework that captures factors influencing consumer behavior within online food retail environments. It also details the methodology for framework development and refinement—a process that identified and integrated evidence across multiple fields of study. The conceptual framework captures both consumer- and retailer-level influences across the entire Path to Purchase as well as the broader social, community, and policy context. Important static attributes of retailer policies and practices and consumer characteristics, preferences, and past behaviors are captured. The framework also emphasizes the dynamic attributes of the online platform, including those of personalized marketing by retailers, customization of the website content, navigation by the consumer, and the two-way interaction between these domains that enables a variety of online food retail interfaces uniquely tailored to consumer preferences.

This conceptual framework makes an important contribution to our understanding of the burgeoning field of online food retail. It serves as a foundation for a deeper study into the influences on consumer food purchase behaviors within online platforms and the interactions between them. To our knowledge, this is the first time that the relatively under-studied domains of personalization and customization of the online food retail environment or the domains of equity and transparency or the social, community, and the policy context, have been considered in any framework. Further investigation is certainly warranted. Future studies could use the framework to compare brick-and-mortar retailers and their online platforms to identify the convergence and divergence of consumer behaviors and retailer responses within these settings. The conceptual framework itself could be empirically tested to support its validity and better establish a hierarchy between attributes. Previous work has used structural equation modelling techniques to examine the hypothesized relationships between constructs in a proposed model and identify possible causal relationships between them [32]. While online retail platforms seem largely similar across contexts, a multi-country investigation of the framework’s domains and their interactions would help confirm its applicability to different settings. Testing the framework among a diverse group of consumers to ensure that it adequately captures their selection and purchase experiences is also warranted.

The conceptual framework provides a foundation for understanding how a lack of transparency within online retail platforms could impact health equity. Indeed, if an equity lens is not applied in the development and implementation of retailer policies, online platforms may unintentionally widen disparities in healthy food access, affordability, and diet quality for vulnerable groups [33]. For instance, lack of transparency around membership fees, costs associated with platform access or delivery, accepted forms of payment, or inappropriate product substitutions for SNAP beneficiaries, may be more detrimental to a household with scarce resources to spend on food than to a household with greater financial resources. Concerns have also been raised about the surveillance practices, data collection, use, storage, sharing, and privacy measures of online retailers [34]. Current practices leverage digital tools to capture sensitive data on consumer purchases, location, and preferences or require people to share personal information to avail of savings [34]. This may facilitate targeted marketing on the basis of race, ethnicity, or socioeconomic status. Although targeted marketing is not harmful in and of itself, it may exacerbate existing disparities in diet-related chronic diseases if used to disproportionately advertise unhealthy products.

The framework may help to study the effect of predatory marketing tactics, similar to those employed across other digital media where communities of color are targeted with the least nutritious products [35]. Indeed, several forms of discriminatory and disparate advertising to vulnerable groups have been identified [34], exposure to which is likely to encourage unhealthy purchases. More research to identify and address these equity gaps is crucial to safeguarding the sub-groups that are disproportionately affected by adverse health outcomes and those that stand to benefit most from policy, systems, and environmental interventions that promote healthy eating.

Finally, the conceptual framework could be used to inform and evaluate public health interventions aimed at improving consumer food choices in the online food environment. On the policy and practice front, the framework could inform: (i) recommendations and standards for best practices related to online food marketing; (ii) specific guidance for online retailers to ensure policy transparency, equitable access, and assurance of privacy; (iii) tailored educational content for consumers unfamiliar with online grocery retail; and (iv) personalized nutrition education and communication. For example, nutrition interventions delivered via the online retail platform could offer personalized healthy shopping lists that draw on information about consumer preferences and budget constraints, offer personalized healthy recipes or meal solutions, or develop a personalized labeling campaign that makes specific nutritional attributes of a product more salient at the point of purchase. SNAP-Ed—SNAP’s voluntary nutrition education program—could partner with online retailers to allow participants to interact with a registered dietitian in real time while food shopping. Local WIC agencies could work with online retailers to create a WIC-friendly web interface with WIC-eligible products, label products as being part of WIC food packages, and allow only WIC-approved substitutions in case of stock-outs.

This study does have its limitations. Despite a comprehensive approach to development, the resulting conceptual framework may not have captured all the elements of the online food retail environment. The descriptions of the constructs in the framework serve as examples and are not exhaustive. The literature reviewed was almost entirely from the US and Europe. Pilot testing and content validity were established for the US context. Therefore, it is possible that the framework may not adequately capture the online environments in other contexts and would need further testing to gauge applicability in different settings, including its applicability to food purchases made via mobile applications. Advances in technology will result in new and innovative features that will need to be incorporated into the evolving conceptual framework.

This study has several strengths. It leveraged multiple methods in the development and refinement of the framework, including key informant interviews, comprehensive systematic literature reviews, mock shopping exercises and group discussions, improving its validity. The framework development drew from multiple disciplines and benefited greatly from the insights of experts across different fields, allowing for an in-depth understanding of the factors influencing consumer purchases and underscoring the need for the public health community to collaborate with scientists and policymakers from non-traditional public health disciplines to map the influence of the online food environment. Finally, applying a public health perspective to the development of the framework expanded its utility in informing future interventions in this field.

## 5. Conclusions

This paper integrated multiple perspectives across a wide range of fields to develop a framework capturing both consumer- and retailer-level factors influencing consumer purchases in the online food retail environment, as well as the broader social, community, and policy context. It identifies important static factors and emphasizes the dynamic nature of personalized marketing by retailers and customizable website content. Equity and transparency in retailer policies and practices are also captured. Researchers, retailers, advocates and policymakers are encouraged to utilize the framework to guide the development and evaluation of interventions, policies, and practices in the online food retail space.

## Figures and Tables

**Table 1:** Description of consumer- and retailer-level domains and constructs that influence consumer behavior in the online food retail environment. EBT, Electronic Benefits Transfer, GMO, genetically modified organism.

**Consumer-Level Attributes**		
**Domain** ***Stage of Path to Purchase*** **(Static vs. Dynamic)**	**Construct**	**Description and Examples**
**Consumer Characteristics** **Preferences and Past Behaviors** *Pre-Shop* *Online Shopping* *Pick-up/Delivery* *Post-Shop* (Static)	Technology Acceptance	Associated factors include familiarity with internet technology, perceived ease of use, risks associated with online food retail, concerns with web security and privacy of personal and financial information, past exposure to online food retail, safe and reliable access to the internet
	Individual and Household Demographics	Factors include age, sex, income, education level, employment, disability, injuries resulting from accidents, urban/rural residence, geographic location, distance from physical store, price sensitivity, marital status, family culture, household size, and social class
	Food-Related Preferences and Behaviors	Associated factors include familiarity with product, health consciousness, perceived need to inspect item or assess sensory properties prior to purchase, dietary preferences and allergies, perceived safety of delivered foods, perceived costs, and value of online versus in-store products
	Attitudes and Beliefs	These encompass consumer beliefs about the online retailer’s brand image, online service quality, perceived importance of social contact while grocery shopping, time savings, convenience, perceived cognitive effort versus gain, perceived comparative advantage compared to brick-and-mortar stores
**Retailer-Level Attributes**		
**Domain** ***Stage of Path to Purchase*** **(Static vs. Dynamic)**	**Construct**	**Description and Examples**
**Retailer Policies and Practices** *Pre-Shop* (Static)	Site Access	Associated policies include ease of initiating and terminating grocery purchase services (online sign-up policies, membership cancellation procedures), referral schemes and incentives that allow risk-free trial of the online platform, multi-channel presence to help streamline access to food retailer services through mobile applications, websites, and the physical store
	Privacy and Data Sharing	These policies determine how consumer data on purchase patterns, personal demographic information, etc., are stored, protected, and used by the retailer and/or shared with third parties
	Inventory Management	Associated practices encompass product availability, assortment and variety of fresh and packaged goods, variety among the brands stocked, similarity in online–offline assortment, product price point, dynamic pricing strategies, similarity in online–offline pricing and promotions
	Collection and Payment	Policies cover consumer-friendly pick-up, delivery, and payment (no cost delivery, pick-up options, waiver of fees for purchases over a designated value or those made at a certain frequency, etc.), multi-option payment channels, acceptance of EBT, loyalty programs linked to redeemable rewards
**Retailer Policies and Practices** *Pick-up/Delivery* *Post-Shop* (Static)	Order Fulfillment	Policies determine the concordance between items delivered compared to items ordered, item quantity, freshness, and the physical condition in which the grocery items arrive, packaging of products, handling of stock-outs, and appropriateness and price of product substitutions
	Order Delivery	Policies cover flexibility in choosing type of collection (click-and-collect versus delivery), availability of delivery slots, length of delivery slot, delivery coordination, the ability to track the real-time location of the groceries purchased, convenient and safe drop-off location options (doorstep delivery, key locations within the community)
	Returns and Order Cancellation	Associated policies address unsatisfactory deliveries, incorrect orders, cancelled orders, and requests for refunds or store credit
**Personalized Marketing by Retailers** *Online Shopping* (Dynamic)	Product—Product Mix	These include the variety, brands, and assortment of products the consumer can view on the online platform
	Price—Discounts	Examples include lower prices on targeted products (discounts, two-for-one deals, cost-saving strategies) which may be open to all customers or exclusive to members of loyalty programs
	Price—Rewards	Rewards include links to coupons, loyalty programs, membership rewards, and other redeemable rewards
	Price—Time-Limited Deals	These include special deals that are valid for a set period (24 h, 3 h, etc.) or weekly flyers meant to incentivize food purchase within a specific period of time
	Placement—Cross-Promotions	Examples include marketing of complementary products anchored to a previous search or to items already in the shopping cart (milk and eggs suggested on a search results page for bread or milk suggested at checkout when cereal is in the shopping cart)
	Placement—Search Result Order	Examples include non-random presentation of products (search results ordered by the most expensive products or display of sponsored products before other items)
	Placement—Recommendations	Examples include seasonal products, popular items, recently viewed products, suggestions based on past purchases, recommended product/brand swaps, or impulse buys (cookies or candy recommended at checkout)
	Promotions—Advertisements	These include products on paid banner advertisements or title cards (large panel of images or text at the top of a page) displayed on the website that link to a separate landing page featuring the sponsored product
	Promotions—Branded Site Content	Examples include branded products integrated into the existing site content, like department images (branded cereal displayed to indicate the breakfast cereal department), branded recipes or meal solutions (branded marinara sauce depicted in a lasagna recipe), promoted product swaps, and retailer-generated shopping lists
	Promotions—User Feedback	This includes highlighting consumer product reviews and ratings to promote the selection of certain products
	Promotions—Social Media	Examples include links to the retailer’s Instagram, Facebook, or other social media pages promoting specific brands or products and opportunities for consumers to share purchased products through personal social media accounts
	Promotions—Point-of-purchase Information	These include labels, nutrient and health claims (non-GMO, whole-grain), and other product descriptors (product source, organic) that may be personalized to promote the selection of certain products
**Customization of Website by Consumer** *Online Shopping* (Dynamic)	Product Information Display	Functional features on the webpage may allow consumers to filter products based on pre-selected information about their allergens, ingredients, nutrition facts, nutrition rating systems, country of origin, product reviews, and ratings based on their preferences
	Site Navigation	Examples include tools and tutorials to help consumers navigate the website, browse through departments, engage with available features to customize the ‘look and feel’ of their online shopping interface (change display size, image size, orientation)
	Shopping Tools	These tools increase the convenience of product search and selection by allowing consumers to choose their preferred setting to create and save shopping lists, notes and wish lists, and allow for product/brand comparisons
**Other Food Marketing** *Pre-Shop* *Online Shopping* *Pick-up/Delivery* *Post-Shop* (Dynamic)	Promotional Strategies	Examples include advertisements, sponsorship, endorsements, search result optimization
	Social Media Strategies	These include strategies that utilize social media content, podcasts, videos, or user-generated content
	Immersive Strategies	These include strategies like advergames, interactive advertisements to increase the marketing that the consumer is exposed to, while increasing consumer site engagement

## References

[1] Business Insider (2020). Online Grocery Shopping Report 2020: Market Stats and Delivery Trends for Ecommerce Groceries. https://www.businessinsider.com/online-grocery-report.

[2] The Nielsen Company & Food Marketing Institute (2017). The Digitally Engaged Food Shopper. www.fmi.org/digital-shopper.

[3] (2018). Nielsen Company & Food Marketing Institute: 70% of Consumers will be Grocery Shopping Online by 2024. https://www.nielsen.com/us/en/press-releases/2018/fmi-and-nielsen-online-grocery-shopping-is-quickly-approaching-saturation/.

[4] Grocery Drive (2020). Online Grocery Reaches New Heights in April. https://www.grocerydive.com/news/online-grocery-reaches-new-heights-in-april/576993/.

[5] Food Navigator (2020). Online Grocery Sales Surge 37% in April to $5.3bn, Finds Brick Meets Click. https://www.foodnavigator-usa.com/Article/2020/05/05/US-online-grocery-sales-surge-37-to-5.3bn-in-April-finds-Brick-Meets-Click#.

[6] Superfood (2020). Online Grocery Shopping Statistics: Pre and Post Covid-19. https://superfood.digital/online-grocery-store-ecommerce-statistics/.

[7] USDA (2020). Supplemental Nutrition Assistance Program Online Purchasing Pilot. https://www.fns.usda.gov/snap/online-purchasing-pilot.

[8] Brookings (2020). For Millions of Low-Income Seniors, Coronavirus Is a Food-Security Issue. https://www.brookings.edu/blog/the-avenue/2020/03/16/for-millions-of-low-income-seniors-coronavirus-is-a-food-security-issue/.

[9] USDA (2020). SNAP Online Purchasing to Cover 90% of Households. https://www.usda.gov/media/press-releases/2020/05/20/snap-online-purchasing-cover-90-households.

[10] National WIC Association (2020). WIC/EWIC Pickup and Delivery Options. https://s3.amazonaws.com/aws.upl/nwica.org/fy20_nwa_factsheet_pickup-and-delivery-requirements_phase-1.pdf.

[11] Glanz K., Bader M.D., Iyer S. (2012). Retail grocery store marketing strategies and obesity: An integrative review. Am. J. Prev. Med..

[12] Dawson J. (2013). Retailer activity in shaping food choice. Food Qual. Pref..

[13] Hand C., Riley F.D.O., Harris P., Singh J., Rettie R. (2009). Online grocery shopping: The influence of situational factors. Eur. J. Mark..

[14] Hansen T. (2005). Consumer adoption of online grocery buying: A discriminant analysis. Int. J. Retail Distrib. Manag..

[15] Cetina I., Munthiu M.-C., Radulescu V. (2012). Psychological and social factors that influence online consumer behavior. Soc. Behav. Sci..

[16] Zheng Q., Chen J., Zhang R., Wang H.H. (2020). What factors affect Chinese consumers’ online grocery shopping? Product attributes, e-vendor characteristics and consumer perceptions. China Agric. Econ. Rev..

[17] Inman J.J., Nikolova H. (2017). Shopper-facing retail technology: A retailer adoption decision framework incorporating shopper attitudes and privacy concerns. J. Retail..

[18] Pauzi S.F.F., Thoo A.C., Tan L.C., Muharam F.M., Talib N.A. (2017). Factors influencing consumers intention for online grocery shopping—A proposed framework. IOP Conf. Ser. Mater. Sci. Eng..

[19] Esbjerg L., Jensen B.B., Bech-Larsen T., de Barcellos M.D., Boztug Y., Grunert K.G. (2012). An integrative conceptual framework for analyzing customer satisfaction with shopping trip experiences in grocery retailing. J. Retail. Cons. Serv..

[20] Darley W.K., Blankson C., Luethge D.J. (2010). Toward an integrated framework for online consumer behavior and decision-making process. A review. Psychol. Mark..

[21] Nguyen D.H., de Leeuw S., Dullaert W.E. (2018). Consumer behaviour and order fulfilment in online retailing: A systematic review. Int. J. Manag. Rev..

[22] Jabareen Y. (2009). Building a conceptual framework: Philosophy, definitions, and procedure. Int. J. Qual. Methods.

[23] Armstrong G., Adam S., Denize S., Kotler P. (2014). Principles of Marketing.

[24] Dominici G. (2009). From marketing mix to e-marketing mix: A literature overview and classification. Int. J. Bus. Manag..

[25] Davis F.D. (1989). Perceived usefulness, perceived ease of use, and user acceptance of information technology. MIS Q..

[26] Cheung C.M., Chan G.W., Limayem M. (2005). A critical review of online consumer behavior: Empirical research. J. Electron. Commerce Org..

[27] Marreiros C., Ness M. (2009). A Conceptual Framework of Consumer Food Choice Behaviour.

[28] Penim J.M.C.D.S. (2013). Online Grocery Shopping: An Exploratory Study of Consumer Decision Making Processes. Masters’s Thesis.

[29] Kurnia S., Chien J.A.W. The Acceptance of The Online Grocery Shopping. Proceedings of the 16th Bled Electronic Commerce Conference.

[30] Glanz K., Johnson L., Yaroch A.L., Phillips M., Ayala G.X., Davis E.L. (2016). Measures of retail food store environments and sales: Review and implications for healthy eating initiatives. J. Nutr. Ed. Behav..

[31] Bronfenbrenner U. (1979). The Ecology of Human Development.

[32] Choi Y.A. (2013). Structural Equation Model of the Determinants of Repeat Purchase Behaviour of Online Grocery Shoppers in the UK. Ph.D. Thesis.

[33] Kumanyika S.K. (2019). A framework for increasing equity impact in obesity prevention. Am. J. Public Health.

[34] Center for Digital Democracy (2020). USDA Online Buying Program for Snap Participants Threatens Their Privacy and Can Exacerbate Racial and Health Inequities, Says New Report. https://www.democraticmedia.org/article/usda-online-buying-program-snap-participants-threatens-their-privacy-and-can-exacerbate.

[35] The Rudd Centre for Food Policy and Obesity (2019). Increasing Disparities in Unhealthy Food Advertising Targeted to Hispanic and Black Youth. http://uconnruddcenter.org/files/Pdfs/TargetedMarketingReport2019.pdf.

